# Hospice or Hospital: The Costs of Dying of Cancer in the Oncology Care Model

**DOI:** 10.1089/pmr.2020.0023

**Published:** 2020-06-25

**Authors:** J. Russell Hoverman, B. Brooke Mann, Sara Phu, Philip Nelson, Jad E. Hayes, Cynthia B. Taniguchi, Marcus A. Neubauer

**Affiliations:** ^1^ U.S. Oncology Network, Texas Oncology, Dallas, Texas, USA.; ^2^ McKesson Corp., The Woodlands, Texas, USA.

**Keywords:** cancer chemotherapy, cost of care at end of life, hospice oncology, palliative oncology

## Abstract

***Background:*** End-of-life management is a difficult aspect of cancer care. With the oncology care model (OCM), we have data to assess both clinical outcomes and total cost of care (TCOC).

***Objective:*** To measure and characterize the TCOC for those who received less than three days of hospice care (HC) at the end of life compared with those who received three days or more.

***Design:*** Assess data on costs and site and date of death from Medicare claims on patients identified in the OCM who received chemotherapy in the six months before death. Standard statistical methods were used to characterize both populations.

***Setting/Subjects:*** Subjects were Medicare patients with cancer who died while managed by U.S. oncology practices in the OCM. Measurements were TCOC in 30-day intervals for the last months of life, cost by site of care at the end of life, and demographic characteristics of the population and association with HC.

***Results:*** There were 7329 deaths. Dying in the hospital was twice the cost of dying at home under HC ($20,113 vs. $10,803). Of demographic groups measured, only black race and a lymphoma diagnosis had <50% hospice enrollment for three days or more before death.

***Conclusions:*** This study reinforces previous studies regarding costs in the last 30 days of life. The graphic representation highlights the dollar cost and the costs of lost opportunity. Using these data to improve communication, addressing socioeconomic support, and formal palliative care integration are potential strategies to improve care.

## Introduction

The cancer community, including patients, families, and providers, are of two minds when it comes to making decisions at the end of life. This dichotomy can be represented by the following two competing narratives. (The patients in these narratives are abstractions from many patients and do not represent any single patient's story.)

Narrative #1: S.G. was a 75-year-old gentleman with lung cancer. After a brief response with combined therapy including immunotherapy, he was started on second line therapy. After two cycles he had lost weight and was short of breath. His physician suggested his disease may be progressing. On discussing options, the patient stated he did not want to “give up.” They chose a third line of chemotherapy. Within days he was admitted to the hospital with fever and profound weakness. After discharge to a rehabilitation facility, he developed fever and respiratory failure, was readmitted, and died in the hospital.

Narrative #2: W.H. was an 85-year-old woman with metastatic colon cancer. She tried standard first line therapy but stopped due to side effects. She tried a clinical trial (“maybe it will help someone”) but again stopped due to toxicity. She was adamant that she did not want to spend resources on heroic measures and on only what was needed to keep her comfortable. She enrolled in hospice care (HC) and died at home six weeks later.

These narratives are both examples of patients doing what they think is “everything,” or at least, not doing “nothing.” The dichotomy between hospital and hospice was demonstrated by a recent report that showed that, for the first time in over half a century, more patients died at home than in the hospital.^1^ Even with this apparent cultural change, an end-of-life discussion remains one of the most difficult aspects of cancer care.

In 2016, funded by provisions in the Affordable Care Act, the Centers for Medicare and Medicaid Innovation began a program with Medicare called the oncology care model (OCM). The intention was to provide support for practices to examine and improve their processes to meet the goals of improving quality and reducing costs. A chief metric was to increase the use of HC at the end of life.^2,3^ With the OCM, oncology providers have access to all claims data associated with their Medicare cancer patients. The U.S. Oncology Network (USON) had 16 practices in 13 states participating in the OCM. The data from the first two years of the program (2016–2018) on 7329 patients are presented here to display the costs of care in the last six months of life, and especially in the last 30 days, to better inform our providers and patients as these decisions are made and give insight into how to improve care.

## Methods

Centers for Medicare and Medicaid Services provided claims data and demographic details, including date of death, for OCM-enrolled patients attributed to USON practices. We measured 30-day interval OCM episode expenditures for *n* = 7329 deceased patients, as well as patient demographics, clinical outcomes (hospice, hospital/nonintensive care unit [ICU], ICU/hospital, and emergency room visits), and treatment categories in the last 30 days. The population was divided into two cohorts: those with three days or more of HC and those with fewer than three days or no hospice care (NOHC). The three-day differentiation is based on data that hospice for less than three days is no better than NOHC. This metric is validated by the National Quality Forum (NQF).^4^ Expenditures were compared between HC and NOHC at the univariate level using a Mann–Whitney U test; categorical variables were compared using chi-squared tests. Multivariate regression was used to determine the association of HC with expenditures in the 30 days before death (EXP30) adjusted for demographic and disease factors.

To identify each patient's place of death, we first evaluated discharge status codes on hospice claims where the discharge date was the same as the patient's date of death. If hospice claims were not available (e.g., in cases wherein the patient did not die while in HC), then we evaluated revenue center codes and patient discharge status codes from any available inpatient claims wherein the discharge date was the same as the patient's date of death. If neither hospice claims nor inpatient claims yielded a place of death, the patient's place of death was categorized as “unknown.”

## Results

**FIG. 1.**
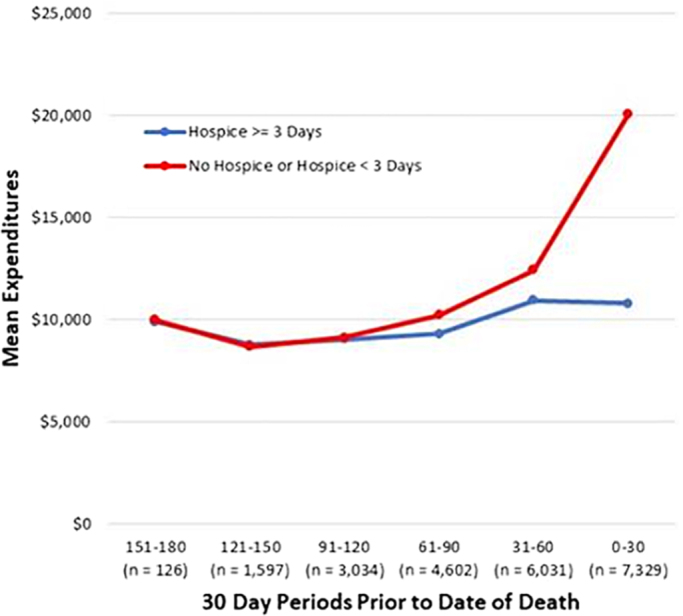
Cost per 30-day period leading up to death.

Of 50,569 individual patients, there were 7329 deaths (14.5%). HC had mean last EXP30 reduction of −$5,641 versus NOHC (95% confidence interval −$6,009 to −$5,252) adjusted for demographics and disease. HC had lower rates of death in hospital (0% vs. 42.1%, *p* < 0.0001). Mean expense by days before death was as follows: NOHC values 0–30 days = $20,113; 31–60 days = $12,438; and 61–90 days = $10,258; and HC values 0–30 days = $10,803; 31–60 days = $10,960; and 61–90 days = $9,311. These data are presented in graphic format and Figure 1 shows the average for both groups of patients over time, illustrating a difference of ∼$10,000 in the last 30 days. Table 1 summarizes hospice versus nonhospice use by demographic group. Table 2 gives expenditures by hospice status and place of death. The total cost of care for episodes ending in death was $318.6 million with the cost of the last 30 days accounting for 34.2% ($109 million) of total cost. All categories of care (hospital, drugs, professional visits, laboratories imaging, etc.), with the exception of hospice, were higher in the NOHC cohort.

**Table 1.** Characteristics of Each Population

Demographics	HC	NOHC	HC (%)	NOHC (%)	p
Total	4121	3208	56.2	43.8	
Age (years)					
<60	167	174	49.0	51.0	0.017
60–69	1022	814	55.7	44.3	0.681
70–79	1816	1415	56.2	43.8	0.797
≥80	1116	805	58.1	41.9	0.127
Gender					
Male	2115	1762	54.6	45.4	0.007
Female	2006	1446	58.1	41.9	0.007
Cancer types					
Breast	455	385	54.2	45.8	0.352
Colon	320	241	57.0	43.0	0.735
Lung	1006	756	57.1	42.9	0.561
Lymphoma	159	217	42.3	57.7	<0.001
Prostate	336	231	59.3	40.7	0.253
Other	1845	1378	57.2	42.8	0.239
Race					
Other or unknown	172	146	54.1	45.9	0.586
White	3338	2472	57.5	42.5	<0.001
Black	249	296	45.7	54.3	< 0.001
Hispanic	281	220	56.1	43.9	0.796
Asian/Pacific Islander	63	60	51.2	48.8	0.422
American Indian/Alaska Native	18	14	56.3	43.8	0.798

HC, hospice care three days or more; NOHC, no hospice care less than three days.

**Table 2.** Expenditures by Hospice Status and Place of Death

Setting	HC	NOHC	Total	% of total	Average final 30-day spend
Hospice—home	3034	390	3424	46.7	$10,098
Hospice—medical facility	1066	506	1572	21.4	$17,481
Hospice—setting unknown	21	9	30	0.4	$13,325
Hospital	0	399	399	5.4	$22,410
ICU	0	950	950	13.0	$28,301
SNF	0	4	4	0.1	$19,400
Unknown	0	950	950	13.0	$11,246
Total	4121	3208	7329	100.0	$14,878

ICU, intensive care unit; SNF, skilled nursing facility.

## Discussion

The population of this study is unique in that these are only cancer patients who have received treatment within the last six months, the population is representative of the population of the country, there are data for different racial and ethnic groups, there are disease-specific data, and the treatment options include current drug choices. The costs associated with the metric of hospice for three days or more (HC) are also presented in a cancer population for the first time. A critical value of this study is the graphic representation of the trajectory of end-of-life costs and potential lost opportunities depending on treatment choices, particularly HC. The current experience with ICU deaths in the coronavirus (COVID-19) epidemic and the pervasive unwanted specter of dying alone make the choices depicted by these data even more compelling.

The overall rate of hospice admission was 56.2%. The demographic data confirm previous studies in that black patients generally have lower hospice use rates (45.7%) than other racial groups, and those with hematologic malignancies also have lower rates (42.3%). Each group, black patients and those with lymphoma, has a significant proportion of patients who chose HC. In the case of race, there may be opportunities to address socioeconomic issues both late in disease^5^ but before hospice admission, and to reduce hospice disenrollment shortly before death.^6^ There is no racial group where the majority would choose to die in the hospital.^7^ In the case of hematologic malignancies, this may be an opportunity to target palliative care involvement.^8–11^ The demographic differences do not alone determine decisions.

Obermeyer et al.,^12^ using an extensive retrospective Medicare database, showed increased costs in the last month of life for patients with poor prognosis cancer. Gidwani-Marszowshi et al.,^13^ in a 98% male Veterans Administration database, demonstrated that costs with HC were substantially lower and associated with much less aggressive care than NOHC. Chastek et al.,^14^ in a commercially insured population, reported similar cost findings. These studies, and others, consistently find that aggressive end-of-life care is more costly without an improvement in survival and is associated with a demonstrated detrimental impact on the satisfaction and well-being of caregivers and family.^15^ This study also shows high cost for those dying in a hospital, particularly in the ICU. There is likely a disproportionate number of black patients and those with heme malignancies and hospice duration of less than three days who die in hospital-based hospices.^16^

The course of a cancer treatment is usually described as a representation of the bell-shaped curve skewed to the right with a survival tail—“a median survival of eight months with a two-year survival of 20% and a five-year survival of 5%,” for example. Physicians and the cancer community are comfortable with pursuing improbable events—moderate to high risk with high reward. However, the community is not well equipped to identify highly improbable events based on age, fitness, comorbidity, tumor responses, line of therapy, and previous toxicities, for example.^17,18^ The profession and the public are enamored with the tail of the curve (the five-year survival of 5%). This image was brought to the public domain by Stephen J. Gould's article^19^—The Median is not the Message—where his own experience as a young man with peritoneal sarcoma argued for pursuing improbable outcomes. However, these bell-shaped curves do not represent the potential nonmonetary costs of treatment. The “hockey-stick” shape of the graph presented in Figure 1 depicts not only dollar costs but also the costs of lost opportunity. There is loss of the opportunity to maximize comfort, to make choices to be at home, to affirm relationships, to reduce the burden on caregivers and family, and to say goodbyes.^20^

The cautionary tale from this image is to be aware of the costs of the one surgery or one dose of chemotherapy too many that leads to dying in the hospital or ICU. Dr. Gould was very aware of the outcomes of pursuing highly improbable choices. Twenty-eight years after his initial episode with sarcoma, he was diagnosed with lung cancer. He was older and the disease had spread widely. He died in his library at home surrounded by his family and his books.^21^

These data can provide benchmarks using the OCM (NQF) hospice metric for assessing the success of end-of-life programs. They also suggest support where socioeconomic conditions place barriers to HC and suggest areas where formal palliative care evaluation could inform decision making.

There are limitations to this study. The cause of death is unspecified and we cannot be sure of the direct relation to cancer. There may be regional or practice cultural differences that impact the use of HC. However, the consistency of the data suggests the trajectory of the curve is likely to be little changed. Also, the place of death for 13% of the population is unknown. Given the approximation of cost for this group to the HC group, it may be that many of these died at home, either unexpectedly or with the care of family.

Within the USON, the change in payment structure and the continual review of quality metrics have focused our attention on end-of-life care. We have developed communication tools to begin an assessment of values early in the course of disease.^22^ The network has emphasized improved communication with patients with more follow-up and symptom management calls, and internally with team building and systematic review of high-risk patients.^23^ A promising aspect of the OCM is the interrogation of data at multiple sites both in and out of USON to uncover and share best practices.^24^ Hospice utilization has increased over the course of the OCM but much remains to be done.

## Abbreviations Used

EXP30: expenditures in the 30 days before death
HC: hospice care
ICU: intensive care unit
NOHC: no hospice care
NQF: National Quality Forum
OCM: oncology care model
SNF: skilled nursing facility
TCOC: total cost of care
USON: U.S. Oncology Network
